# Personality Traits and Attention in Israeli Older Adults: An Exploratory Study

**DOI:** 10.3390/geriatrics11030060

**Published:** 2026-05-18

**Authors:** Dubi Lufi, Iris Haimov

**Affiliations:** Department of Psychology, The Center for Psychobiological Research, The Max Stern Yezreel Valley College, D.N. Emek Yezreel 1930600, Israel; dubilufi@gmail.com

**Keywords:** attention, personality, older adults, computerized test, big five inventory

## Abstract

**Background/Objectives:** The purpose of the present study was to assess the relationships between attention level and personality traits among 44 older adults aged from 65 to 75 in the Israeli population. The participants were 19 females and 25 males, with a mean age of 68.13 years, and 12.32 years average of formal education, who provided clinical history for screening. **Methods:** The participants completed the 44-item Big Five Inventory, the d2 Test of Attention, and the Mathematics Continuous Performance Test (MATH-CPT), a computerized test that measures participants’ attention levels by recording their responses to visual stimuli. **Results:** The results showed significant correlations between attention and the domains of Conscientiousness and Neuroticism. Linear regression analysis, with the MATH-CPT measures as the dependent variable and the Big Five personality factors as independent variables, revealed a significant model explaining 41.60% of the variance in MATH-CPT performance. Linear regression analysis of the measures of the d2 test of attention as dependent variables and the Big Five factors as independent variables showed a significant model that explained 47.70% of the variance. No association was found between age and the domains of the Big Five personality traits. **Conclusions:** This study discusses the implications of the significant correlations between personality domains and measures of attention in older adults. The findings suggest that individuals who are organized, task-oriented, goal-focused, responsible, imaginative, creative, and interested in educational experiences may be better able to perform attention-related tasks in older age.

## 1. Introduction

A perennial argument is whether personality can be changed, how and when it can be changed, and how stable it is. The most common opinion is that personality is a trait that can be modified well into adulthood, as it is connected to educational investments, responsive to parental behavior, and influenced by policy interventions [1]. Others have considered personality to be a stable trait [2]. This understanding of personality is supported by Cattell’s conception [3] that personality is like plaster, which will never soften or change after the age of 30. Recent findings have contradicted the plaster theory (Roberts, Walton, & Viechtbauer, 2006 [4]; Roberts & Mroczek, 2008 [5]).

When one considers the decline of personality domains in older adults, a different picture may appear when we compare that decline to other changes in personality. Hedden and Gabrieli [6] summarized their review of the decline in older age by stating the following:

Both cross-sectional and longitudinal studies find robust declines in abilities such as encoding new memories of episodes or facts, working memory (the simultaneous short-term maintenance and manipulation of information involving executive processes) and processing speed (the speed with which information can be processed). By contrast, short-term memory (a component process of working memory), autobiographical memory, semantic knowledge and emotional processing remain relatively stable.

Others [7] who researched changes among personality traits at various ages found relatively stable personality changes when comparing adolescents to adults, and stronger correlations among older adults aged 70 and above. In other words, the correlations between personality traits reflected small changes at younger ages and even fewer changes among older adults. In considering the change in personality traits in old age and its influence on economic behavior, Cobb-Clark and Schurer [8] found that personality traits remain stable among working-age adults, while the changes in the Big Five Inventory were found to be small not only among adults but across all age groups. Their findings are similar to those of Bouchard and Loehlin [9] who found that 40 to 60 percent of the traits measured by the Big Five Inventory remained stable throughout the life cycle.

Recent studies have revealed changes in personality in older adults. Cipriani, Debbio, and Fiorino [10], as well as [11], found that Conscientiousness reduced the risk of developing dementia, while Neuroticism increased the risk of developing dementia. By contrast, the same studies reported that Agreeableness and Extraversion were not associated with dementia. In a longitudinal study of 10 years in Japan, Ref. [12] concluded their extensive research with the following statement: “The results showed that higher Openness to Experience was associated with a reduction in risk for cognitive decline. In post hoc analyses of individuals with mild cognitive deficits, Conscientiousness predicted lower risk for severe cognitive decline. Neuroticism, Extraversion, and Agreeableness were not associated with changes in global cognitive status” (p. 23).

The connection between personality and attention has been a popular area of research for the last 40 years. Using the Big Five Inventory, Ref. [13] claimed that Extraversion predicted increased attentional performance, while Neuroticism was associated with decreased attention control. Another study utilized meta-analysis to show that introverts performed better in sustained attention tasks but were poorer in maintaining efficiency over time [14]. These findings rely on Eysenck’s theory [15], which linked vigilance to arousal and claimed that introverts perform better than extroverts on tasks requiring the maintenance of attention over time.

Cognitive ability and personality traits are different constructs. In normal healthy aging, cognitive functions (for example, memory, attention and executive functions) decline steadily, while personality traits remain mostly steady. Significantly, big shifts in personality characteristically occur when there is a cognitive impairment. Terracciano et al. [16] assessed Big Five traits and found minimal changes before dementia starts, but speeds up when the disease accelerates significantly during impairment. It may mean that normal age-related concentration (attention) does not match with personality deterioration, while personality gives the impression of being relatively unaffected. Only when pathological aging processes occur (e.g., cognitive impairment) do characteristic maladaptive personality profiles emerge (e.g., lower discipline or higher anxiety). In this study, intelligence was not directly measured but was inferred from participants’ educational level and their ability to complete high school. Overall, these findings suggest that personality may influence cognitive aging; however, cognitive decline does not necessarily correspond to changes in personality among healthy older adults.

Attention is a multifaceted concept; it is one of the main cognitive functions that allows people to select, keep, and regulate information processing in regular daily functioning. There are various models to distinguish attention. A common model of attention is the following division: Selective attention; Sustained attention; Divided attention; and Executive attention [17]. A different approach to define attention is found in the DSM-5 [18] where a clinical symptom domain is used. Here, attention is defined as attention deficit hyperactivity disorder (ADHD) [18] which is a neurodevelopmental disorder with disorder in attention and hyperactivity/impulsivity. Here, the disorder is defined through a checklist of attentional behaviors without a separate theoretical definition of attention. In the present research we based our work on the definition of the DSM-5.

Personality reflects stable individual differences in forms of affect, cognition, and behavior. Attentional control states that the ability to freely control attention, including inhibiting distracting information, preserving focus over time, and willingly shifting attention when task demands are needed [19]. The Big Five model hypothesizes personality using five traits: Neuroticism, Extraversion, Conscientiousness, Openness to Experience, and Agreeableness [20]. Within this outline, attentional control can be seen as a proximal process that mediates the impact of these traits on visible behavior.

Few researchers have examined the relationships between personality variables and measures of attention among older participants. Still, there are several studies in this field dealing with the association between personality and various areas of cognitive and emotional functioning. Research has found that Conscientiousness correlates with healthy behaviors such as weight control, healthier diet, and non-smoking. In addition, higher Conscientiousness helps to preserve cognitive maintenance in older adults, mainly among individuals with mild cognitive deficits [21,22].

Curtis, Windsor, and Soubelet [23] and Franchow, Suchy, Thorgusen [24] and Williams [22] claimed that Openness to Experience can contribute to increased cognitive reserve among older adults. In addition, Carver and Connor-Smith [25] and Penley and Tomaka [26] postulated that Openness to Experience can reduce cognitive decline when combined with cognitively stimulating activity, similar to coping with stress and/or emotional response. Furthermore, Openness to Experience was found to be closely associated with participation in cognitive activities, but did not have a positive influence on changes in cognitive functioning [27].

The purpose of the present research was to address the gap in research exploring the relationships between personality traits and attention in older adults. The basic goal of the research was exploratory: to find which type of personality has the highest correlation with attention. Based on past research, it was hypothesized that the domains of Conscientiousness and Openness to Experience would show the highest correlation between attention and personality.

## 2. Material and Method

### 2.1. Participants

Forty-four Israeli participants between the ages of 65–75 (mean age = 68.13 years, SD = 3.4), including 19 females and 25 males, volunteered to participate in the study. The participants were recruited through advertisements and talks given at local senior centers. Participants were asked to complete clinical history questions. Inclusion criteria, based on responses to these questions, were as follows: (a) sufficient knowledge of Hebrew to complete the Big Five Inventory; and being Israeli citizen speaking Hebrew all their life, and having at least high school education; (b) no current regular use of psychiatric medications; (c) no psychiatric or neurological disorders; and (d average intelligence was not directly measured but inferred via education and functioning of completing high school education. The participants’ mean educational level was 12.32 years of formal education (SD = 1.74). Of those willing to take part in the research, 95% were successful in the inclusion criteria and completed the tasks of the research.

### 2.2. Materials

The Mathematics Continuous Performance Test (The MATH-CPT) [28] (Lufi & Fichman, 2012) is a computerized CPT-type test designed to assess attention. The MATH-CPT uses a sequence of 450 simple mathematical problems projected on a computer screen to serve as a visual stimulus. These problems involve addition, subtraction, multiplication, and division. During the test, one problem appeared on the screen together with a result that could be right or wrong (e.g., 1 + 4 = 5; or 4 × 2 = 7); the answer was never greater than 9. The participants observed one problem at a time on the computer screen and had to decide whether the solution was correct or incorrect, pressing ‘1’ for a correct answer or ‘2’ for an incorrect answer. The test lasted approximately 10 to 20 min depending on the reaction time of each participant. During construction of the MATH-CPT [28], test–retest reliability after one week of testing with the main measures used in the MATH-CPT indicated an average correlation of 0.73 for the test’s main measures. A lower score in the following variables indicates better performance (Attention Score; Reaction Time; SD Time; and Impulsivity). In addition, a discriminant function analysis was used to compare a control group without attention deficit hyperactivity disorder (ADHD) to a group with ADHD. The results indicated that the test could correctly identify 90.8 percent of the participants in both groups. The reason the MATH-CPT was chosen for the study over other commonly used CPTs is that it seems to fit better a population of adults, and has been used in research with older adults [29,30].

The d2 Test of Attention [31] is a paper-and-pencil test of cancellation aimed at assessing attention. The test involves a task in which participants are asked to cancel specific designated letters (*p* or *d*) with small vertical lines above and/or below the letters. The test has 14 rows of 47 characters in each row—a total of 658 characters. The person who is given the test is allowed 20 s to cancel the designated letter (the letter *d*) in each row, with 2 small vertical lines below and/or above the letter. The task lasted 4 min and 40 s. Before starting the test, each participant was given a full explanation and a chance to practice the task in a one-line trial. The test assessed attention through the following variables: rate of cancellation, accuracy, consistency of work, number of mistakes, and three formulas derived from the results of the test. The d2 Test of Attention has been used in many studies to assess attention, and has included a test–retest reliability of >0.90 in numerous studies [31]. The validation of the d2 test has shown significant correlations with other measures of attention.

The Big Five Inventory [32]; Hebrew translation of the 44-item version by Etzion & Laski [33]. Based on Cattel’s 16 personality factors [27], this test is also known as the five-factor model (FFM). This is one of the most-used personality inventories, and it has been replicated cross-culturally and in various contexts [33,34]. The validity and reliability of the inventory have been proven in numerous studies, some of which are mentioned below.

The questionnaire uses the following five factors (dimensions or domains) to describe the personality of the person who answers the inventory. Extraversion describes a person who is sociable, optimistic, outgoing, talkative, and sensation seeking, and is associated with longer lives [35]. Agreeableness describes a person who is agreeable, generally well-tempered, friendly, cooperative with others, trustworthy, and not suspicious towards others, and is associated with longer lives [35]. Conscientiousness describes a person who is responsible, has a tendency to be well-organized, task-focused in tracking goals, and is the strongest predictor of long life as compared to the other Big Five factors [35]. Neuroticism describes a person with a tendency towards proneness to unpleasant experiences, e.g., anxiety, anger, and depression, and refers to the person’s degree of emotional stability; it is associated with shorter lives [35]. Openness to Experience describes a person who tends to be imaginative, interested in culture and educational experiences, and creative; it is associated with longer lives [35]. Each factor is then further divided into personality facets. Scoring is performed by means of a 5-point Likert scale where 1 = Disagree Strongly and 5 = Agree Strongly. For this study, we used the brief 44-item version, which was aimed to yield an estimate of the five factors originally assessed in the long version.

### 2.3. Procedure

Informed consent was obtained from the participants. In addition, they were informed that they could stop their participation in the study at any time they wished. The participants gave their written consent to participate in the study of their own free will and without any financial benefits. The participants were tested individually at their homes or in a quiet room at the assisted living facility where they resided. The geographical location of the study was in Northern Israel. First, they answered the short clinical history questions (to ensure appropriate cognitive ability to perform the required tasks of the research), followed by the Big Five Inventory. Then the research assistant administered the d2 test of attention, with a line of practice preceding the test itself. Next, a practice of 30 sample problems of the MATH-CPT was administered to every participant who took the test, after which they responded to the MATH-CPT.

### 2.4. Data Analyses

SPSS (Version 21) software was used for statistical analyses. Gender differences were assessed by an independent sample *t*-test. Pearson Correlations were used to identify relationships between the different variables used in the study. Linear Regression with an Enter procedure was used to find the weight of the relationships among the personality variables and the measures of attention. Significance was set at *p* = 0.05.

Although the primary hypotheses focused on Conscientiousness and Openness, all Big Five personality traits were included in the regression analyses. This approach was chosen due to the exploratory nature of the study and the relatively limited research examining these relationships in older adults. Including all five traits allowed for a more comprehensive examination of their unique and shared contributions to attention performance.

We evaluated regression assumptions for all models. Multicollinearity diagnostics indicated no concerns (VIFs ranged from 1.22 to 1.41). Residual plots supported linearity. Shapiro–Wilk tests indicated mild deviations from normality in some models; however, given the robustness of OLS to modest non-normality and the absence of extreme outliers, results were considered reliable. For the d2 model, the Breusch–Pagan test indicated heteroscedasticity; therefore, coefficients were re-estimated using heteroscedasticity-consistent (HC3) robust standard errors, which did not materially alter the pattern of results.

## 3. Results

Comparison of gender differences showed no statistically significant differences in any of the study variables, allowing the researchers to continue data analysis without consideration of possible gender differences.

As noted in the table footnotes, lower scores on several MATH-CPT and the d2 indices reflect better attentional performance. Accordingly, the negative correlations and regression coefficients (with Conscientiousness, for example) indicate superior attention among individuals higher on this trait. Table 1 presents the means and standard deviations of all the variables used in the study.

Table 2 presents three significant correlations between the MATH-CPT measures and the Big Five personality traits. In addition, a significant correlation was found between age and reaction time (RT) in the MATH-CPT. Eight significant correlations were identified between the Big Five Inventory and the d2 Test of Attention. No significant correlations were observed between age and the Big Five personality traits or the measures of the d2 Test of attention.

Table 3 presents a multiple linear regression analysis examining whether personality traits (Extraversion, Agreeableness, Conscientiousness, Neuroticism, Openness), age, and years of education predicted final attention scores on the MATH-CPT. The overall model was statistically significant (F_(7, 33)_ = 3.37, *p* = 0.01). The model explained 41.60% of the variance. Two variables were significant: Conscientiousness and Openness to Experience. Conscientiousness emerged as a significant negative predictor (β = −0.52, *p* = 0.01), whereas openness was a significant positive predictor (β = 0.43, *p* = 0.01). No other predictors reached statistical significance.

In addition, Table 3 presents a multiple linear regression analysis examining whether personality traits (Extraversion, Agreeableness, Conscientiousness, Neuroticism, Openness), age, and years of education predict concentration performance as measured by the d2 Test of Attention. The overall regression model was statistically significant (F_(7, 33)_ = 3.39, *p* = 0.01), explaining 47.70% of the variance. In this analysis, Conscientiousness and Extraversion were significant predictors. Conscientiousness was a positive predictor (β = 0.34, *p* = 0.01), whereas Extraversion was a negative predictor (β = −0.40, *p* = 0.01). No other predictors reached statistical significance.

Table 4 presents a linear regression analysis (Enter method) examining the effect of the five personality variables on age. The model was not statistically significant (F(5, 39) = 0.67, *p* = 0.65), explaining 8% of the variance.

## 4. Discussion

The present research was intended to find the relationships between attention levels and personality factors among individuals aged 65–75 years. Two tests of attention, a computerized test (the MATH-CPT) and a paper-and-pencil test (the d2 Test of Attention), were used in the study, along with one measure of personality, the Big Five Inventory. The results showed low to moderate correlations between the measures of attention and the Big Five Inventory factors; the most significant correlations were found with the factors of Conscientiousness, and Neuroticism. Linear regression strengthened these findings when these personality domains and the main measure assessed by the MATH-CPT, the Attention Score, explained 41.60 percent of the variance between personality and attention. In addition, the measure of Concentration Performance of the d2 test of Attention and the measure of the Big Five Inventory explained 47.70 percent of the variance between measures of attention on the d2 Test of Attention and the variables of the Big Five Inventory. In addition, a linear regression assessment of the five personality variables as independent variables and their effect on age, within the narrow age range of 65 to 75 years, as the dependent variable yielded a non-significant result.

Conscientiousness showed consistent associations with attention performance across both computerized (MATH-CPT) and paper-and-pencil (d2) results. Higher levels of Conscientiousness were associated with better attentional efficacy, reflected in a faster rate of response, lower impulsivity, fewer errors, and higher concentration and overall performance scores. These associations remained significant after controlling for multiple comparisons using the false discovery rate (FDR) procedure, indicating a robust and convergent pattern across attention modalities.

The interpretation of these findings is that a person who is well organized, task-oriented and focused on their goals, responsible, and more competent at attention-related tasks, imaginative, creative, and interested in culture and educational experiences will be more capable of performing attention-related tasks in older adults. These findings are similar in some ways to the findings of [36], who reached their results with a younger population. MacLean and Arnell indicated that Openness to Experience predicted higher target accuracy; however, they found that Conscientiousness predicted lower overall target accuracy and less cognitive flexibility. Considering that Conscientiousness is arguably the most important Big Five Inventory domain in the prediction of health outcomes, one can predict the longevity and health of older adults who score high on Conscientiousness. This claim is strengthened by the possibility that personality is correlated with health outcomes in a similar manner as cognitive measures, and in some cases even more [1].

Results showing that the Big Five factors did not correlate with age require additional consideration. Like various cognitive functions which showed a reduction in performance as participants aged, i.e., spatial orientation, inductive reasoning, verbal memory, and perceptual speed [37], attention also showed a decline in ability with age [32,33]. Unlike these functions, it is encouraging to discover that personality traits did not show a significant decline. These findings were found in the present study by means of the Big Five Inventory; however, similar findings are likely for other personality measures as the Big Five factors are representative of personality traits as a whole. The positive side of this finding is that although aging is associated with the reduction in many physical, mental, cognitive, and emotional aspects, personality traits remain relatively intact. This may help older adults to perform well in personal and social interactions, socialize more often, and enjoy their lives more, even if they experience reductions in other aspects of their performance.

This study has several limitations that should be considered when interpreting the findings. First, the sample was limited to a single age group (65–75 years), which restricts the ability to examine developmental differences across the broader spectrum of late adulthood. Second, although correlations with age were examined, the cross-sectional design does not allow for conclusions regarding intra-individual changes over time or causal relationships between personality and attentional processes. Third, the relatively small sample size, while selected to ensure a homogeneous and cognitively healthy group, limits statistical power and the generalizability of the findings. Finally, the use of a single cultural group further constrains the external validity of the results, as personality–cognition relationships may vary across sociocultural contexts. Future research should address these limitations by employing larger, more diverse samples, longitudinal designs, and cross-cultural comparisons to better understand the dynamics between personality and attention in aging populations.

## 5. Conclusions

Research assessing the population of older adults has become very popular in recent years. Most studies are performed in an attempt to better understand the nature of the decline in various aspects of human functioning, such as physical, cognitive, social, personal, and emotional. The knowledge acquired so far has been significant. In addition, scientists are trying to discover ways to attenuate or slow down the decline in these human functions. Our study confirmed previous studies that have revealed a correlation between personality domains and attention, specifically among older adults. Our findings showed that personality traits previously considered to have a positive correlation with health and personal functioning are also associated with higher attention levels. In this sense, the famous slogan “a sound mind in a sound body” could be rephrased as “a sound cognition in a sound body.” Positive personality traits may influence cognitive traits, and as such may contribute to the longevity and quality of life of older adults.

## Figures and Tables

**Table 1:** Means and Standard Deviation of All Variables Used in the Study (N = 44).

Questionnaire	Variable	Mean	Standard Deviation
Big Five Inventory	Extraversion	24.93	4.37
	Agreeableness	35.41	5.03
	Conscientiousness	34.89	4.82
	Neuroticism	19.91	6.20
	Openness	31.89	5.90
MATH-CPT	Attention Score ^a^	−0.48	1.10
	RT (Min.) ^a^	14.72	4.03
	SD Time ^a^	1.12	0.87
	Impulsivity ^a^	9.00	8.75
	Correct Responses	434.86	13.41
d2 Test of Attention	Concentration Performance	2.60	62.19
	Overall Performance	211.24	78.52
	Percentage of Errors ^a^	49.78	22.49
	Correct Responses	68.08	36.28

^a^ = For these variables, lower scores indicate better attentional performance. Notes: RT = time taken to respond to the test; SD Time = variability of the RT; Impulsivity = a combination of the number of anticipatory responses faster than 500 milliseconds and the fast incorrect responses, answered faster than the average response time; Correct Responses = total number of correct responses.

**Table 2:** Pearson Inter-correlations of Attentional and Personality measures with Age (N = 44).

		Big Five Inventory Domains					
	Variable	Extraversion	Agreeableness	Conscientiousness	Neuroticism	Openness	Age
MATH-CPT	Attention Score ^a^	0.07	0.04	−0.37 *	0.19	0.26	−0.19
	RT (Sec.) ^a^	0.14	−0.03	−0.30 *	0.19	0.21	−0.40 **
	SD Time ^a^	0.31	0.09	−0.16	−0.08	0.20	−0.19
	Impulsivity ^a^	−0.03	0.01	−0.34 *	0.10	0.24	−0.7
	Correct Responses	0.04	−0.06	0.27	−0.03	−0.24	0.8
Age		0.05	−0.17	0.21	0.08	0.10	
d2 Test of Attention	Concentration performance	−0.20	0.13	0.40 *	0.36 *	−0.13	0.25
	Overall performance	−0.16	0.08	0.36 *	0.38 *	−0.05	0.02
	Percentage of errors ^a^	0.18	−0.06	−0.43 **	0.39 *	0.06	0.24
	Correct responses	0.16	0.04	0.42 *	0.39 *	−0.01	0.09

* *p* < 0.05, ** *p* < 0.01. ^a^ = For these variables, lower scores indicate better attentional performance; therefore, negative coefficients reflect superior performance. Notes: RT **=** Time taken to respond to the test; SD Time = variability of the RT; Impulsivity = a combination of the number of anticipatory responses faster than 500 milliseconds and the fast incorrect responses, answered faster than the average response time; Correct Responses = total number of correct responses. Notes: To control for multiple comparisons, *p*-values were adjusted using the Benjamini–Hochberg false discovery rate (FDR) procedure, applied separately for each attention test table.

**Table 3:** Linear regression analysis of Big Five variables, MATH-CPT, Age, Years of Education, and d2 Performance.

Variable	B	Std. Error	Beta	t	Significance
Final Attention Score (MATH-CPT) as dependent variable					
Conscientiousness	0.11	0.04	0.52	3.14	0.04
Openness	0.08	0.03	0.43	2.92	0.05
Agreeableness	0.04	0.04	0.04	1.24	0.22
Extraversion ^a^	−0.01	0.04	0.04	−2.00	0.84
Neuroticism	0.03	0.03	0.03	0.91	0.37
Age	0.05	0.03	0.06	1.83	0.08
Years of education	0.02	0.10	0.03	0.16	0.87
Concentration Performance (d2) as dependent variable					
Conscientiousness	4.80	2.36	0.34	2.03	0.05
Extraversion ^a^	−5.83	2.72	−0.40	2.14	0.04
Neuroticism ^a^	−3.16	1.63	−0.31	1.82	0.08
Openness ^a^	−0.12	1.70	−0.01	−0.07	0.94
Agreeableness	−1.36	2.41	0.10	−0.57	0.57
Age	−1.63	1.95	−1.13	−0.83	0.41
Years of Education	11.50	6.36	2.90	1.74	0.09

^a^ = For these variables, lower scores indicate better attentional performance; therefore, negative coefficients reflect superior performance.

**Table 4:** Linear regression of Big Five Variables Predicting Age.

Variable	B	Std. Error	Beta	t	Significance
Agreeableness ^a^	−0.12	0.19	−0.11	0.63	0.53
Neuroticism ^a^	−0.07	0.16	−0.08	0.41	0.68
Extraversion ^a^	−0.09	0.22	−0.08	0.44	0.67
Conscientiousness ^a^	−0.26	0.20	−0.23	1.26	0.21
Openness	0.15	0.16	0.17	0.96	0.34

^a^ = For these variables, lower scores indicate better attentional performance; therefore, negative coefficients reflect superior performance.

## Data Availability

The data presented in this study are available on request from the corresponding author due to privacy and ethical restrictions.

## Abbreviations

The following abbreviations are used in this manuscript:

CPT	Continuous Performance Test
MATH-CPT	Mathematics Continuous Performance Test
ADHD	Attention Deficit Hyperactivity Disorder
FFM	Five-Factor Model

## References

[1] Almlund M., Duckworth A.L., Heckman J.J., Kautz T., Hanushek E., Machin S., Wößmann L. (2011). Personality psychology and economics. Handbook of the Economics of Education Handbook of the Economics of Education.

[2] Roberts B.W. (2009). Back to the Future. J. Pers. Ass. JRP.

[3] Cattell M. (1890). Ment Test Meas. Mind.

[4] Roberts B.W., Walton E., Viechtbauer W. (2006). Patterns of Mean-Level Change in Personality Traits across the Life Course: A Meta-Analysis of Longitudinal Studies. Psychol. Bull..

[5] Roberts B.W., Mroczek D. (2008). Personality Trait Change in Adulthood. Curr. Res. Psychol. Sci..

[6] Hedden T., Gabrieli J.D.E. (2004). Insights into the ageing mind: A view from cognitive Neuroscience. Nat. Rev. Neurosci..

[7] Klimstra T.A., Bleidorn W., Asendorpf J.B., van Aken M.A., Denissen J.J. (2013). Correlated change of Big Five personality traits across the lifespan: A search for determinants. J. Res. Personal..

[8] Cobb-Clark D.A., Schurer S. (2011). The stability of big-five personality traits. Econ. Lett..

[9] Bouchard T.J., Loehlin J.C. (2001). Genes, Evolution and Personality. Behav. Gen..

[10] Cipriani G.G., Debbio B.A.D., Di Fiorino M. (2015). Personality and dementia. J. Nerv. Ment. Dis..

[11] Low L.F., Lackersteen H.S.M. (2013). Does personality affect risk for dementia? A systematic review and meta-analysis. Am. J. Geriat. Psychiatry.

[12] Nishita Y., Tange C., Tomida M., Otsuka R., Ando F., Shimokata H. (2016). Personality and global cognitive decline in Japanese community-dwelling elderly people: A 10-year longitudinal study. J. Psychosom. Res..

[13] Hahn S., Buttaccio D.R., Hahn J., Lee T. (2015). Personality and attention: Levels of neuroticism and extraversion can predict attentional performance during a change detection task. Q. J. Exp. Psychol..

[14] Koelega H.S. (1992). Extraversion and vigilance performance: 30 years of inconsistencies. Psychol. Bull..

[15] Eysenck H.J. (1967). The Biological Basis of Personality.

[16] Terracciano A., Luchetti M., Stephan Y., Löckenhoff C.E., Ledermann T., Sutin A.R. (2023). Changes in personality before and during cognitive impairment. J. Am. Med. Dir. Assoc..

[17] Salthouse T.A. (2010). Major Issues in Cognitive Aging.

[18] American Psychiatric Association (2013). Diagnostic and Statistical Manual of Disorders.

[19] Posner M.I., Rothbart M.K. (2007). Research on attention networks as a model for integration of psychological science. Annu. Rev. Psychol..

[20] McCrae R.R., Costa P.T., John O.P., Robins R.W., Pervin L.A. (2008). The five-factor theory of personality. Handbook of Personality: Theory and Research.

[21] Luchetti M., Terracciano A., Stephan Y., Sutin A.R. (2015). Personality and cognitive decline in older adults: Data from a longitudinal sample and meta-analysis. J. Gerontol. B Psychol. Sci. Soc. Sci..

[22] Williams P.G., Suchy Y., Kraybill M.L. (2013). Preliminary evidence for low openness to experience as a pre-clinical marker of incipient cognitive decline in older adults. J. Res. Personal..

[23] Curtis R.G., Windsor T.D., Soubelet A. (2015). The relationship between Big-5 personality traits and cognitive ability in older adults—A review. Aging Neuropsychol. Cogn..

[24] Franchow E., Suchy Y., Thorgusen S.R., Williams P. (2013). More than education: Openness to experience contributes to cognitive reserve in older adulthood. Aging Sci..

[25] Carver C.S., Connor-Smith J. (2010). Personality and Coping. Annu. Rev. Psychol..

[26] Penley J.A., Tomaka J. (2002). Associations among the Big Five, emotional responses, and coping with acute stress. Pers. Individ. Differ..

[27] Bielak A.A., Anstey K.J., Christensen H., Windsor T.D. (2012). Activity engagement is related to level, but not change in cognitive ability across adulthood. Psychol. Aging J..

[28] Lufi D., Fichman N. (2012). The development and use of a computerized test, MATH-CPT, to assess attention. Percept. Mot. Ski..

[29] Lufi D., Haimov I. (2018). Effects of age on attention level: Changes in performance between the ages of 12 and 90. Aging Neuropsychol. Cogn..

[30] Lufi D., Segev S., Blum A., Rosen T., Haimov I. (2015). The Effect of Age on Attention Level: A Comparison of Two Age Groups. Int. J. Aging Hum. Dev..

[31] Brickenkamp R., Zillmer E. (1998). The d2 Test of Attention.

[32] John O.P., Donahue E.M., Kentle R.L. (1991). The “Big Five” Inventory—Versions 4a and Technical Report.

[33] Etzion D., Laski S. (1998). Hebrew Version of the Big Five Inventory.

[34] Cattell R.B. (1943). The description of personality: Basic traits resolved into clusters. J. Abnorm. Soc. Psychol..

[35] Roberts B.W., Kuncel N.R., Shiner R., Caspi A., Goldberg L.R. (2007). The Power of Personality: The Comparative Validity of Personality Traits, Socioeconomic Status, and Cognitive Ability for Predicting Important Life Outcomes. Perspect. Psychol. Sci..

[36] MacLean M.H., Arnell K.M. (2010). Personality predicts temporal attention costs in the attentional blink paradigm. Psychon. Bull. Rev..

[37] Schaie K.W. (1996). Intellectual Development in Adulthood: The Seattle Longitudinal Study.

